# Supplementary material for: A Likelihood Approach for Real-Time Calibration of Stochastic Compartmental Epidemic Models
Source: PLoS Comput Biol. 2017 Jan 17;13(1):e1005257. doi: 10.1371/journal.pcbi.1005257 (PMC5240920; doi:10.1371/journal.pcbi.1005257)
Supplement: S1 File — (TAR.GZ) [file pcbi.1005257.s014.tar.gz › HSPH_Online-SI-Revision/IPoi09/n10-extreme/n10-extreme_BENCH_table-8.pdf]

| | | | | | | | | | | | | | | | | | | | | | | | | | | | |
|---|---|---|---|---|---|---|---|---|---|---|---|---|---|---|---|---|---|---|---|---|---|---|---|---|---|---|---|
| 1 | R0 | {2.68692, 12.5884} | {2.11256, 8.46842} | {{1.38889, 3.83841}, {2.84915, 12.8842}} | {23.6679, 35.3644} | inf | 21. | 65.8929 | {4., 163.} | 243.707 | 0.57656 | newly | 13. | 87.625 | {4., 241.} | 594.918 | 0.807963 | Reff | 2.6759 | 2.22822 | {1.46693, 2.84231} | 19.4441 | ar | 9089. | 7776.39 | {4886., 9298.} | 15.5325 |
| 2 | R0 | {1.79802, 12.5412} | {1.28086, 3.45796} | {{1.25474, 3.26445}, {1.26656, 3.29313}} | {28.8267, 72.4272} | inf | 125. | 162.5 | {11., 287.} | 73.6 | 0.424429 | newly | 89. | 336.333 | {18., 579.} | 299.813 | 0.69918 | Reff | 1.74857 | 1.16744 | {1.02625, 1.26083} | 33.235 | ar | 7412. | 3823.67 | {3364., 4777.} | 48.4125 |
| 3 | R0 | {2.6174, 10.1105} | {1.95419, 11.8373} | {{1.14639, 6.01426}, {2.688, 17.5607}} | {27.2208, 37.6832} | inf | 4. | 2.12 | {0., 5.} | 80.5 | 0.781855 | newly | 3. | 2.45 | {0., 6.} | 109. | 0.900501 | Reff | 2.61609 | 1.95354 | {1.14628, 2.68719} | 27.2115 | ar | 9109. | 3818.58 | {1., 8989.} | 58.3943 |
| 4 | R0 | {2.27532, 14.3149} | {2.15195, 11.8056} | {{1.67405, 7.28757}, {2.8568, 18.2812}} | {18.357, 29.8232} | inf | 174. | 211.556 | {25., 475.} | 82.5032 | 0.392518 | newly | 107. | 188.463 | {19., 480.} | 123.139 | 0.410742 | Reff | 2.20251 | 2.14449 | {1.49811, 2.81017} | 19.7337 | ar | 8478. | 8148.72 | {6810., 9320.} | 10.619 |
| 5 | R0 | {2.96649, 18.0674} | {1.72112, 5.9869} | {{1.30094, 3.04996}, {2.59133, 11.8519}} | {42.0032, 66.8635} | inf | 107. | 278.851 | {2., 780.} | 214.735 | 0.695605 | newly | 64. | 330.064 | {3., 810.} | 441.456 | 0.798852 | Reff | 2.92051 | 1.84325 | {1.25992, 2.68979} | 36.8859 | ar | 9476. | 6296.47 | {61., 9342.} | 33.5535 |
| 6 | R0 | {2.94778, 16.0002} | {2.23452, 7.62113} | {{1.43579, 3.38828}, {2.91946, 10.9639}} | {24.2788, 52.3685} | inf | 56. | 55.283 | {8., 122.} | 63.2749 | 0.446203 | newly | 41. | 92.4528 | {11., 197.} | 160.009 | 0.512387 | Reff | 2.92125 | 2.31687 | {1.72724, 2.894} | 20.7762 | ar | 9321. | 7978.28 | {6300., 9324.} | 14.5547 |
| 7 | R0 | {2.17562, 11.7184} | {1.85741, 8.17929} | {{1.07662, 2.14605}, {2.78007, 15.1048}} | {27.5978, 44.5463} | inf | 23. | 15.9153 | {0., 42.} | 72.8077 | 0.818863 | newly | 18. | 24.1525 | {0., 93.} | 129.284 | 0.86787 | Reff | 2.16844 | 2.0055 | {1.07608, 2.88811} | 23.9322 | ar | 8345. | 5440.27 | {3., 9324.} | 40.2244 |
| 8 | R0 | {2.7904, 17.8966} | {2.40194, 12.2788} | {{1.83586, 7.55154}, {2.85582, 15.7559}} | {15.0037, 31.414} | inf | 98. | 338.803 | {38., 745.} | 272.363 | 0.564807 | newly | 46. | 256.93 | {42., 475.} | 469.994 | 0.689215 | Reff | 2.74575 | 2.29592 | {1.78261, 2.73022} | 16.9068 | ar | 9285. | 8435.8 | {7673., 9318.} | 9.2891 |
| 9 | R0 | {2.51574, 16.376} | {2.46071, 12.7197} | {{1.95355, 8.88568}, {2.85582, 15.963}} | {11.4341, 22.9415} | inf | 112. | 191.677 | {25., 471.} | 119.409 | 0.402132 | newly | 55. | 154.185 | {25., 384.} | 207.189 | 0.449381 | Reff | 2.47171 | 2.39361 | {1.90967, 2.79388} | 11.4436 | ar | 8937. | 8650.29 | {7881., 9304.} | 5.99892 |
| 10 | R0 | {2.56405, 18.1787} | {1.75253, 6.75051} | {{1.30094, 3.11158}, {2.44101, 11.6597}} | {32.5004, 62.8658} | inf | 124. | 349.471 | {79., 657.} | 201.913 | 0.509233 | newly | 69. | 437.882 | {114., 799.} | 546.49 | 0.794735 | Reff | 2.51226 | 1.7079 | {1.12331, 2.28793} | 32.4176 | ar | 9005. | 6987.57 | {4696., 8914.} | 22.7018 |
| 11 | R0 | {2.475, 17.1709} | {1.82571, 14.2222} | {{1.52414, 9.52864}, {2.12588, 19.2116}} | {26.2339, 20.6161} | inf | 144. | 166.061 | {17., 358.} | 72.4632 | 0.392425 | newly | 77. | 97.0816 | {9., 202.} | 80.0424 | 0.410826 | Reff | 2.41461 | 1.77938 | {1.46226, 2.04953} | 26.3077 | ar | 8870. | 7359.78 | {5954., 8325.} | 17.0262 |
| 12 | R0 | {2.14974, 11.8619} | {2.21109, 12.5033} | {{1.3618, 4.98002}, {2.88523, 18.2127}} | {19.5501, 32.2275} | inf | 45. | 51.8571 | {4., 123.} | 81.0582 | 0.446533 | newly | 37. | 46.2698 | {2., 99.} | 93.1789 | 0.475709 | Reff | 2.13147 | 2.22435 | {1.45124, 2.83791} | 17.0112 | ar | 8414. | 8057.3 | {5445., 9302.} | 11.2575 |
| 13 | R0 | {2.20385, 14.4767} | {2.10823, 15.0858} | {{1.76659, 10.896}, {2.46477, 19.4475}} | {11.8075, 22.1915} | inf | 171. | 162.839 | {36., 388.} | 64.7974 | 0.398573 | newly | 105. | 93.125 | {17., 203.} | 63.6224 | 0.407925 | Reff | 2.13685 | 2.08623 | {1.71336, 2.39801} | 10.3514 | ar | 8347. | 8080.86 | {7059., 8900.} | 8.25275 |
| 14 | R0 | {2.35671, 13.8357} | {2.25817, 9.34128} | {{1.50263, 4.22172}, {2.91762, 13.3345}} | {19.3969, 32.7} | inf | 81. | 200.759 | {28., 409.} | 176.288 | 0.429611 | newly | 53. | 221.259 | {29., 454.} | 336.532 | 0.558036 | Reff | 2.32631 | 2.3707 | {1.66559, 2.84085} | 15.6215 | ar | 8719. | 8631.69 | {7215., 9340.} | 7.29244 |
| 15 | R0 | {1.91368, 10.1082} | {1.56777, 7.02163} | {{1.25656, 3.82585}, {2.20345, 13.5548}} | {24.1928, 45.2079} | inf | 100. | 143.139 | {14., 297.} | 95.1944 | 0.381293 | newly | 95. | 168.222 | {18., 360.} | 123.86 | 0.406201 | Reff | 1.87579 | 1.62881 | {1.18519, 2.45153} | 22.71 | ar | 7486. | 6270.83 | {3905., 8896.} | 24.4041 |
| 16 | R0 | {2.48727, 14.882} | {2.42074, 13.7586} | {{1.93539, 9.06807}, {2.88523, 17.3228}} | {12.6157, 18.2337} | inf | 30. | 31.0149 | {4., 70.} | 74.3284 | 0.418274 | newly | 19. | 24.8806 | {2., 58.} | 92.2231 | 0.426136 | Reff | 2.47508 | 2.41428 | {1.93176, 2.86341} | 12.3187 | ar | 8894. | 8357.73 | {7457., 9310.} | 9.25058 |
| 17 | R0 | {2.46795, 13.1915} | {1.89715, 7.91946} | {{1.20105, 3.02112}, {2.75274, 14.3523}} | {28.101, 46.1216} | inf | 43. | 7.55932 | {0., 20.} | 82.4202 | 1.19826 | newly | 22. | 12.2034 | {0., 32.} | 84.7458 | 0.956489 | Reff | 2.45487 | 2.09059 | {1.30997, 2.80636} | 21.695 | ar | 8796. | 6015.41 | {2., 9180.} | 33.7289 |
| 18 | R0 | {2.96603, 16.7543} | {2.19602, 8.95303} | {{1.49267, 4.22172}, {2.86001, 13.5542}} | {25.9833, 46.5628} | inf | 59. | 88.7302 | {15., 185.} | 96.2335 | 0.34326 | newly | 37. | 109.667 | {16., 271.} | 216.388 | 0.439546 | Reff | 2.93429 | 2.27737 | {1.58366, 2.80891} | 22.4223 | ar | 9394. | 8300.73 | {6346., 9322.} | 11.6674 |
| 19 | R0 | {2.25216, 9.90465} | {2.18662, 12.1805} | {{1.35081, 5.4396}, {2.85582, 17.9172}} | {21.9151, 42.1357} | inf | 7. | 8.02941 | {0., 23.} | 94.5378 | 0.559095 | newly | 5. | 8.22059 | {0., 22.} | 148.529 | 0.672569 | Reff | 2.24946 | 2.24239 | {1.47549, 2.79805} | 19.1789 | ar | 8631. | 6361.71 | {2., 9260.} | 31.4308 |
| 20 | R0 | {2.70837, 18.1923} | {2.25034, 12.8774} | {{1.42659, 5.32185}, {2.88523, 18.2127}} | {19.3579, 30.0021} | inf | 85. | 47.8615 | {8., 98.} | 52.8869 | 0.460108 | newly | 45. | 40.0462 | {5., 85.} | 59.4872 | 0.410769 | Reff | 2.67506 | 2.28777 | {1.838, 2.89991} | 17.5349 | ar | 9183. | 8196.55 | {7193., 9341.} | 11.3767 |
| 21 | R0 | {2.30288, 15.5877} | {2.17084, 14.6923} | {{1.80156, 10.0119}, {2.64373, 19.7855}} | {11.2361, 17.1237} | inf | 145. | 254.627 | {46., 504.} | 113.594 | 0.381174 | newly | 76. | 149.237 | {31., 311.} | 128.256 | 0.380855 | Reff | 2.2437 | 2.1194 | {1.73733, 2.55014} | 11.7563 | ar | 8674. | 8256.34 | {7453., 9100.} | 7.42214 |
| 22 | R0 | {2.82208, 10.7544} | {1.91498, 11.8662} | {{1.23086, 5.61163}, {2.70836, 18.727}} | {32.7053, 39.4643} | inf | 4. | 1.82 | {0., 4.} | 72. | 0.752403 | newly | 9. | 1.81 | {0., 4.} | 85.2222 | 1.27406 | Reff | 2.82039 | 1.91441 | {1.23074, 2.70809} | 32.6891 | ar | 9333. | 3415.5 | {1., 9028.} | 63.43 |
| 23 | R0 | {1.69923, 10.1366} | {1.29483, 3.07993} | {{1.24751, 2.5364}, {1.31535, 3.47086}} | {23.7988, 69.6158} | inf | 114. | 151.077 | {22., 300.} | 86.9096 | 0.461373 | newly | 88. | 370.346 | {46., 742.} | 338.855 | 0.688015 | Reff | 1.65454 | 1.18496 | {1.01722, 1.36201} | 28.3811 | ar | 7055. | 4230.27 | {3121., 5168.} | 40.0387 |
| 24 | R0 | {2.64944, 19.054} | {2.68448, 17.4448} | {{2.31231, 13.3585}, {2.93978, 19.9817}} | {7.40357, 10.7741} | inf | 333. | 482.629 | {70., 1095.} | 91.5058 | 0.363834 | newly | 143. | 252.914 | {40., 569.} | 113.167 | 0.37992 | Reff | 2.50266 | 2.48609 | {2.13661, 2.83542} | 8.6317 | ar | 9147. | 9127.3 | {8846., 9380.} | 2.10983 |
| 25 | R0 | {2.53191, 10.9214} | {1.59297, 14.0326} | {{1.19018, 7.7153}, {1.99753, 19.2013}} | {37.0843, 40.723} | inf | 6. | 8.19608 | {1., 21.} | 93.4641 | 0.418496 | newly | 9. | 4.47059 | {0., 10.} | 66.4488 | 0.67091 | Reff | 2.52811 | 1.701 | {1.27652, 2.11265} | 32.7167 | ar | 9009. | 5418.02 | {8., 8185.} | 39.8599 |
| 26 | R0 | {1.89155, 6.76944} | {2.3176, 7.17018} | {{1.43579, 3.47086}, {2.9194, 10.1535}} | {30.1759, 30.0416} | inf | 21. | 20.5821 | {0., 49.} | 77.6119 | 0.597073 | newly | 28. | 44.4925 | {0., 110.} | 127.452 | 0.712854 | Reff | 1.88512 | 2.39329 | {1.70913, 2.93624} | 32.2435 | ar | 7348. | 7568.31 | {5., 9391.} | 30.6358 |
| 27 | R0 | {2.63149, 15.6979} | {2.38262, 13.4995} | {{1.75445, 7.55154}, {2.8568, 17.9172}} | {13.851, 20.7327} | inf | 43. | 32.3833 | {2., 74.} | 66.3953 | 0.542436 | newly | 23. | 29.6167 | {1., 92.} | 100.797 | 0.54209 | Reff | 2.61465 | 2.39877 | {1.81519, 2.84411} | 13.258 | ar | 9104. | 8121.68 | {6867., 9304.} | 11.877 |
| 28 | R0 | {2.85141, 12.6992} | {2.0346, 7.08091} | {{1.22544, 2.60601}, {2.79906, 12.6465}} | {28.919, 49.0901} | inf | 11. | 24.99 | {0., 69.} | 249.545 | 1.2616 | newly | 9. | 91.37 | {0., 304.} | 1029.22 | 1.40147 | Reff | 2.84685 | 2.02403 | {1.22532, 2.76665} | 29.1093 | ar | 9317. | 3958.81 | {1., 9132.} | 57.5448 |
| 29 | R0 | {2.54244, 12.9085} | {1.99407, 15.4067} | {{1.5767, 10.5556}, {2.37462, 19.3966}} | {21.5687, 27.0319} | inf | 35. | 28.9672 | {1., 49.} | 60.2342 | 0.539602 | newly | 22. | 15.459 | {0., 29.} | 61.0283 | 0.602028 | Reff | 2.52846 | 2.0432 | {1.67636, 2.3561} | 19.1918 | ar | 9034. | 7223.75 | {6127., 8757.} | 20.0381 |
| 30 | R0 | {2.10982, 11.7636} | {2.60347, 15.5232} | {{2.02235, 10.6804}, {2.96978, 18.8445}} | {25.5527, 36.5111} | inf | 84. | 247.164 | {33., 553.} | 219.403 | 0.453454 | newly | 65. | 151.776 | {25., 368.} | 164.684 | 0.40654 | Reff | 2.07501 | 2.51225 | {1.98766, 2.87031} | 23.7798 | ar | 8224. | 8951.84 | {8044., 9381.} | 10.779 |
| 31 | R0 | {2.76312, 15.5396} | {2.46655, 8.5494} | {{1.90341, 5.7296}, {2.92375, 10.872}} | {13.598, 44.9831} | inf | 70. | 67.5217 | {5., 153.} | 71.0352 | 0.491575 | newly | 46. | 111.449 | {9., 263.} | 180.025 | 0.536929 | Reff | 2.73438 | 2.44704 | {1.88323, 2.89692} | 13.7481 | ar | 9220. | 8210.84 | {7009., 9370.} | 11.6612 |
| 32 | R0 | {2.21727, 15.5439} | {1.98172, 16.6746} | {{1.79436, 13.6186}, {2.1968, 19.9579}} | {10.6252, 13.6213} | inf | 175. | 187.897 | {29., 456.} | 69.4187 | 0.357133 | newly | 111. | 95.6034 | {15., 240.} | 64.0106 | 0.408124 | Reff | 2.14809 | 1.91916 | {1.71419, 2.13815} | 10.712 | ar | 8359. | 7887.26 | {7326., 8378.} | 5.8077 |
| 33 | R0 | {2.72912, 10.8159} | {1.93447, 11.374} | {{1.20105, 3.74835}, {2.60465, 17.2664}} | {29.939, 39.9801} | inf | 10. | 9.10714 | {1., 24.} | 72.5 | 0.49388 | newly | 16. | 7.07143 | {1., 21.} | 70.7589 | 0.764529 | Reff | 2.72393 | 2.13304 | {1.35437, 2.76046} | 22.8565 | ar | 9196. | 6596.84 | {7., 9165.} | 28.5588 |
| 34 | R0 | {2.20928, 10.1308} | {2.26077, 3.17765} | {{1.43152, 1.47098}, {2.96265, 4.56948}} | {19.6357, 68.6337} | inf | 22. | 263.23 | {0., 687.} | 1179.41 | 1.57317 | newly | 14. | 1220.74 | {0., 3562.} | 8699.57 | 2.07802 | Reff | 2.20155 | 2.1394 | {1.41993, 2.82096} | 18.4391 | ar | 8388. | 4997.39 | {1., 9330.} | 46.6272 |
| 35 | R0 | {2.10103, 15.2673} | {2.21862, 11.4804} | {{1.41571, 4.94504}, {2.83341, 16.5525}} | {21.3486, 29.6987} | inf | 193. | 456.05 | {98., 997.} | 159.18 | 0.433385 | newly | 113. | 367.133 | {78., 693.} | 239.853 | 0.521947 | Reff | 2.038 | 2.11338 | {1.36528, 2.69992} | 20.365 | ar | 8264. | 8187.22 | {5730., 9331.} | 12.5163 |
| 36 | R0 | {2.74914, 19.2775} | {2.10197, 14.9434} | {{1.76659, 10.896}, {2.46533, 19.3027}} | {23.5407, 22.7248} | inf | 143. | 152.339 | {24., 317.} | 70.5955 | 0.369328 | newly | 67. | 91.6129 | {14., 182.} | 90.2263 | 0.383943 | Reff | 2.68618 | 2.04949 | {1.70417, 2.41206} | 23.7027 | ar | 9301. | 8081.74 | {7301., 8900.} | 13.1089 |
| 37 | R0 | {2.78161, 16.0136} | {2.07354, 15.1014} | {{1.6568, 10.5556}, {2.46976, 19.391}} | {25.4558, 17.8615} | inf | 38. | 20.9254 | {2., 45.} | 63.7078 | 0.569798 | newly | 25. | 13.4776 | {1., 29.} | 66.6269 | 0.656226 | Reff | 2.76659 | 2.11765 | {1.77142, 2.48699} | 23.4616 | ar | 9240. | 7862.34 | {6888., 8905.} | 14.9191 |
| 38 | R0 | {2.4454, 8.33501} | {1.98558, 13.0352} | {{1.15964, 6.25107}, {2.76203, 18.9248}} | {24.119, 65.8682} | inf | 3. | 5.27397 | {1., 13.} | 130.594 | 0.444452 | newly | 9. | 6.0274 | {0., 10.} | 92.6941 | 0.819974 | Reff | 2.44369 | 2.02489 | {1.15848, 2.75789} | 22.5995 | ar | 8853. | 5207.1 | {1., 9182.} | 42.554 |
| 39 | R0 | {2.9154, 12.3861} | {1.90428, 13.313} | {{1.14884, 8.17412}, {2.59602, 19.2116}} | {34.7218, 27.9166} | inf | 14. | 4.57143 | {1., 9.} | 76.7347 | 0.806518 | newly | 12. | 4.12857 | {0., 6.} | 89.6429 | 1.00565 | Reff | 2.91044 | 1.88617 | {1.20932, 2.57731} | 35.2477 | ar | 9371. | 5074.76 | {1., 8976.} | 45.8507 |
| 40 | R0 | {2.40923, 15.1963} | {2.13731, 8.70894} | {{1.50263, 4.22172}, {2.75598, 12.8589}} | {19.5349, 42.7012} | inf | 149. | 178.436 | {16., 347.} | 77.8401 | 0.422905 | newly | 86. | 212.891 | {43., 462.} | 176.342 | 0.479391 | Reff | 2.34731 | 2.20225 | {1.45565, 2.71161} | 16.9406 | ar | 8735. | 7956.44 | {6113., 9271.} | 13.4679 |
| 41 | R0 | {2.63137, 15.238} | {2.17498, 12.9877} | {{1.42659, 5.32185}, {2.7811, 19.6712}} | {19.6703, 27.4096} | inf | 88. | 129.138 | {17., 298.} | 96.6693 | 0.409229 | newly | 67. | 93.1724 | {8., 232.} | 95.368 | 0.433307 | Reff | 2.59269 | 2.24952 | {1.69308, 2.76212} | 15.6849 | ar | 9150. | 8326.43 | {6935., 9262.} | 9.49218 |
| 42 | R0 | {2.48769, 19.4593} | {2.16076, 17.4898} | {{1.85052, 12.8742}, {2.30371, 19.6625}} | {13.1417, 10.3925} | inf | 353. | 250.343 | {46., 504.} | 53.1479 | 0.395174 | newly | 145. | 120.91 | {15., 232.} | 54.0607 | 0.361648 | Reff | 2.34714 | 2.0791 | {1.82843, 2.24874} | 11.4197 | ar | 8932. | 8373.48 | {7640., 8662.} | 6.25305 |
| 43 | R0 | {2.61199, 10.214} | {1.95419, 11.8373} | {{1.14639, 6.01426}, {2.688, 17.5607}} | {27.128, 36.9974} | inf | 1. | 2.12 | {0., 5.} | 174. | 0.522516 | newly | 2. | 2.45 | {0., 6.} | 137.5 | 0.808934 | Reff | 2.61147 | 1.95354 | {1.14628, 2.68719} | 27.1322 | ar | 9061. | 3818.58 | {1., 8989.} | 58.2693 |
| 44 | R0 | {2.50994, 18.3382} | {2.1744, 17.8105} | {{1.87975, 14.0718}, {2.30371, 19.6625}} | {13.3684, 8.90863} | inf | 194. | 159.354 | {29., 356.} | 58.3981 | 0.378188 | newly | 107. | 79.0769 | {10., 171.} | 59.9425 | 0.440412 | Reff | 2.42761 | 2.12163 | {1.93512, 2.26399} | 12.6045 | ar | 8827. | 8384.98 | {7895., 8629.} | 5.00754 |
| 45 | R0 | {2.44283, 16.8338} | {2.36823, 12.4636} | {{1.739, 7.10524}, {2.93371, 17.1903}} | {14.5879, 26.9658} | inf | 169. | 126.095 | {20., 278.} | 66.3943 | 0.476298 | newly | 105. | 111.683 | {14., 271.} | 78.3522 | 0.42366 | Reff | 2.37394 | 2.29982 | {1.723, 2.78613} | 14.814 | ar | 8878. | 8508.44 | {7136., 9295.} | 7.34829 |
| 46 | R0 | {2.51815, 18.1462} | {2.21628, 14.976} | {{1.88607, 11.3733}, {2.64373, 19.7855}} | {13.8849, 20.6297} | inf | 163. | 1045.68 | {285., 1910.} | 549.476 | 0.731873 | newly | 80. | 527.081 | {160., 872.} | 565.06 | 0.755535 | Reff | 2.44689 | 1.72526 | {1.15155, 2.17586} | 29.8312 | ar | 8978. | 8426.98 | {7817., 9079.} | 6.62282 |
| 47 | R0 | {2.88885, 16.8987} | {2.07437, 14.2265} | {{1.6568, 9.44073}, {2.48947, 19.391}} | {28.1941, 22.3323} | inf | 49. | 45.6415 | {6., 91.} | 68.2326 | 0.427418 | newly | 30. | 29.0189 | {2., 58.} | 71.8239 | 0.487467 | Reff | 2.86458 | 2.1172 | {1.69789, 2.49375} | 26.0904 | ar | 9337. | 7835.89 | {6648., 8899.} | 16.077 |
| 48 | R0 | {2.58709, 13.8786} | {2.07801, 8.26796} | {{1.40116, 4.00571}, {2.85273, 12.8842}} | {23.8862, 41.4051} | inf | 46. | 27.7719 | {1., 61.} | 61.3654 | 0.669423 | newly | 26. | 42.1579 | {0., 106.} | 135.695 | 0.658766 | Reff | 2.57002 | 2.1789 | {1.36472, 2.8586} | 21.011 | ar | 9146. | 7125.79 | {52., 9347.} | 22.8807 |
| 49 | R0 | {2.42959, 10.7132} | {1.98558, 13.0352} | {{1.15964, 6.25107}, {2.76203, 18.9248}} | {23.9157, 38.6911} | inf | 18. | 5.27397 | {1., 13.} | 78.1583 | 0.870689 | newly | 13. | 6.0274 | {0., 10.} | 89.2518 | 0.94948 | Reff | 2.42424 | 2.02489 | {1.15848, 2.75789} | 22.3529 | ar | 8863. | 5207.1 | {1., 9182.} | 42.5755 |
| 50 | R0 | {2.60513, 13.2796} | {1.89096, 10.4627} | {{1.20105, 3.78882}, {2.56757, 17.005}} | {28.4713, 35.8149} | inf | 24. | 19.8077 | {2., 39.} | 58.1731 | 0.440816 | newly | 29. | 19.5577 | {1., 40.} | 66.2467 | 0.607942 | Reff | 2.59445 | 2.05532 | {1.3914, 2.59187} | 22.1023 | ar | 9095. | 7091.58 | {708., 9100.} | 22.2857 |
